# Best Treatment Options for Severe *Helicobacter pylori* Infections

**DOI:** 10.5152/tjg.2025.24543

**Published:** 2025-06-23

**Authors:** Gulhan Kanat Unler, Ozgur Hilal Erinanc, Aydın Karakoca, Huseyin Savas Gokturk

**Affiliations:** 1 Department of Gastroenterology, Başkent University Faculty of Medicine, Konya, Türkiye; 2 Department of Pathology, Baskent University Faculty of Medicine, Konya, Türkiye; 3 Department of Statistics, Necmettin Erbakan University Faculty of Science, Konya, Türkiye

**Keywords:** *H*. *pylori*, eradication regimens, severe colonization

## Abstract

**Background/Aims:**

*Helicobacter pylori* *(H*.* pylori) *affects half of the world’s population. Increasing antibiotic resistance seems to be causing significant clinical problems. The efficacy of bismuth-containing sequential therapy with clarithromycin (BSTC), bismuth-containing sequential therapy with levofloxacin (BSTL), and bismuth-containing quadruple therapy (BQT) regimens on *H*.* pylori* eradication was investigated. The authors also investigated whether high gastric *H*.* pylori* colonization density affected treatment success through different treatment regimens.

**Materials and Methods:**

A total of 751 *H*.* pylori–*positive patients were included retrospectively in the following treatment groups: sequential therapy with clarithromycin, sequential therapy with levofloxacin, and bismuth-containing quadruple therapy.

**Results:**

There was a significant difference between the 3 treatment protocols in terms of treatment success rates. When the success rates of the applied treatments were examined, the highest success rate was BSTL (85.3%), which was statistically significantly higher than BQT (74.8%) and BSTC (74.8%). A significant difference was found between the success rates of the protocols applied in the group with high bacterial density (*P* = .003). The success rates in this group were calculated as BSTL (88.6%), BQT (71.4%), and BSTC (79.4%).

**Conclusion:**

It was concluded that BSTL may be the best option for treating *H*.* pylori* infections in first-line treatment. This regimen is particularly effective in cases of severe *H*.* pylori* colonization.

**Main Points**

- Increasing antibiotic resistance and the decrease in *Helicobacter pylori* (*H*.* pylori*) eradication targets are significant clinical problems.
- A bismuth-containing sequential therapy with a levofloxacin regimen may be preferred for the first-line treatment of *H*.* pylori* infection.
- Bismuth-containing sequential therapy with a levofloxacin regimen is particularly effective in cases of severe *H*.* pylori* colonization.

## Introduction

*Helicobacter pylori* (*H*.* pylori*) is a Gram-negative bacterium that affects nearly half of the world’s population.^1^ **Helicobacter* pylori* infection causes gastritis, peptic ulcer disease, mucosa-associated lymphoid tissue lymphoma, and gastric cancer. It also plays a role in the development of several systemic diseases, including idiopathic thrombocytopenia and iron deficiency anemia.^2^^-^^4^

The prevalence of *H*.* pylori *is 10%-50% in developed countries and 80% in developing countries.^1^ In the TURHEP study, a population cross-sectional study on the prevalence of *H. pylori* infection in Türkiye, the prevalence of *H. pylori* in Türkiye was 82.5%.^5^ This high prevalence of *H*.* pylori *and the burden of associated diseases have made its eradication a challenging issue. ^6^ Guidelines for the treatment of *H*.* pylori* recommend first-line treatment for patients in areas with high clarithromycin resistance with bismuth quadruple therapy for 10-14 days or concurrent quadruple therapy without bismuth. Clarithromycin-containing triple therapy is recommended only in areas with low clarithromycin resistance and only in patients who have not received macrolide antibiotics.^6^^-^^8^

It is important to understand that *H*.* pylori *eradication rates vary among countries, mostly due to differences in antibiotic resistance. Therefore, each country/region should review its own therapeutic results and the effectiveness of various eradication regimens in *H*.* pylori *treatment.^9^Treatments given in the clinic in 2013 in 621 patients with *H*.* pylori *achieved eradication rates using intention to treat (ITT) and per-protocol (PP) analysis by treatment groups of 74.6% and 75.6% in classic quadruple treatment, 70.2% and 70.4% in sequential therapy with clarithromycin, 88.5% and 90.3% in bismuth-enhanced sequential therapy (ST) with clarithromycin, 77.9% and 78.5% in sequential therapy with levofloxacin, and 76.1% and 76.2% in hybrid treatment.^10^

In the present study, the efficacy of bismuth-containing ST with clarithromycin (BSTC), bismuth-containing ST with levofloxacin (BSTL), and bismuth-containing quadruple therapy (BQT) regimens on *H*.* pylori *eradication 10 years later was investigated. The authors investigated whether high gastric *H*.* pylori *colonization density affected treatment success through different treatment regimens.

## Materials and Methods

Ethical approval for this single-center retrospective study was received from Başkent University Ethics Committee (E-9460333604.01-407243/September 12, 2024). Gastroscopy examinations of patients who presented to the clinic with dyspeptic symptoms between January 2022 and August 2024 were evaluated retrospectively. Eight hundred seventy patients who were detected as having *H*.* pylori *from pathology reports and received eradication treatment were included in the study. Histopathologic analysis of endoscopic biopsy specimens was used to identify *H*.* pylori* infection status. *H*.* pylori *density in biopsies taken from the antrum and corpus was evaluated according to the Sydney classification by a single experienced pathologist. The density of *H*.* pylori* colonization was graded as mild, moderate, or severe according to the Sydney classification.^11^If there was a difference between the 2 specimens in terms of density, the highest grade was selected.

A total of 119 patients who had prior unsuccessful empirical *H*.* pylori *eradication therapy, were aged under 18 years, had allergies to antibiotics (amoxicillin, metronidazole, clarithromycin, levofloxacin), were on proton pump inhibitors (PPIs) or H2 antagonists within the last 2 weeks, were on bismuth or antibiotics (amoxicillin, metronidazole, clarithromycin) within the last month, or who had missing medical information (insufficient data about the treatment protocol) were excluded.

Patients were given detailed explanations of the use of the medications and informed about possible adverse effects. They were also given written information about medication use and a physician’s phone number. Proton pump inhibitors were prescribed 20 minutes before meals, and antibiotics and bismuth were prescribed after meals.

There were BSTC, BSTL, and BQT regimens for *H*.* pylori* eradication. Seven hundred fifty-one patients constituted the 3 different groups, as given in Table 1. Six weeks after treatment ended, repeat endoscopies were performed and graded according to the Sydney score as before. Patients were grouped based on successful or failed treatment.

Demographic characteristics were compared between the 2 groups using the Chi-square test. Continuous and categorical variables were analyzed using the Bonferroni test. A *P*-value less than .05 was considered statistically significant.

## Results

A total of 751 patients were included in the study; 728 patients were included in the final analysis because 23 did not complete the treatment.

It was found that the mean ages did not differ between the treatment protocols and that the treatment protocols were homogeneous in terms of age (*P* = .959). The mean age of the patients was 46.49 ± 0.72 (range, 18-75) years. Of the total 751 patients, 425 were female (56.5%) and 326 were male (43.5%). When the homogeneity of the treatment groups in terms of sex was tested, it was found that each treatment group was homogeneous (*P* = .092).

Analyses performed are given for the PP (per-protocol) treatment completed group. Intention to treat results was not sufficient to draw a meaningful conclusion because the number of patients who did not complete the treatment was very small (n = 23). In the treatment groups, 8 patients in the BQT treatment group, 9 in the BSTL group, and 6 patients in the BSTC group could not complete the treatment due to adverse effects. No statistical significance was found between the groups in terms of adverse effect frequency (*P* = .601). The most common adverse effects were diarrhea (n = 10), nausea and vomiting (n = 5), bitter taste in the mouth (n = 3), skin rash (n = 3), and dizziness (n = 2).

There was a significant difference between the 3 treatment protocols in terms of treatment success rates (*P* = .003). When the success rates of the applied treatments were examined, the highest success rate was BSTL (85.3%), which was statistically significantly higher than BQT (74.8%) and BSTC (74.8%). Eradication rates of treatment regimens are given in Table 2.

When the success rates of the applied treatment protocols were examined, it was observed that BSTL treatment success rates showed a significant difference according to sex (*P* = .015). BSTL had a success rate of 81.3% in women and 91.5% in men, significantly higher for men than women. It was observed that the success rates of BQT (*P* = .931) showed no significant difference according to sex, 75% in women and 74.5% in men. BSTC (*P* = .752) showed no significant difference according to sex with success rates of 73.8% in women and 75.7% in men.

When the success rates of the applied treatment protocols were examined to see if they differed according to the endoscopy results, it was found that BSTL (*P* = .885), BQT (*P* = .966), and BSTC (*P* = .286) did not differ according to the endoscopy results.

The authors examined whether the success rates of the treatment protocols applied according to the bacterial density were different. No statistically significant difference was found between the treatment protocols applied in the group with low bacterial density (*P* = .582). A significant difference was found between the success rates of the protocols applied in the group with medium bacterial density (*P* = .022). In this group, the success rates were calculated as BSTL (84.9%), BQT (72.7%), and BSTC (66.2%). A significant difference was found between the success rates of the protocols applied in the group with high bacterial density (*P* = .003). The success rates in this group were calculated as BSTL (88.6%), BQT (71.4%), and BSTC (79.4%). Eradication rates of regimens according to *H*.* pylori *density are given in Table 3.

## Discussion

The increasing prevalence of antibiotic resistance to agents used in *H*. *pylori* treatment complicates managing the infection.^6^The European *H*.* pylori* study suggested that treatment regimens should achieve an eradication rate over 80% on ITT analysis and 85% on PP analysis to be acceptable as first-line therapy for *H*.* pylori* eradication.^12^In general, eradication rates with these treatment protocols are low in the country. Eradication rates of bismuth-containing quadruple regimens in Türkiye vary between 77% and 96.4%, sequential treatments (ST) vary between 39% and 82%, and levofloxacin containing treatments, the eradication rates ranging from 82% to 95%.^13^ According to more recent studies, the eradication rates for concurrent therapy and ST were greater initially but dropped to 80% in the following years.^14^^-^^17^In Türkiye, eradication rates of the BQT regimen were 81.1% in the study by Gokcan et al^18^ and 82.3% in the study by Uygun et al.^19^

In the present study, bismuth-based quadruple therapy, and levofloxacin and clarithromycin STs were compared as first-line treatment for *H*.* pylori* eradication. Bismuth-containing sequential therapy with levofloxacin had the highest eradication rate for *H*.* pylori* (*P* = .003). In addition, the density of *H*.* pylori *colonization was related to eradication success; the *H*.* pylori* eradication rate decreased as the density of *H*.* pylori* colonization increased in BQT and BSTC treatments. Bismuth-containing sequential therapy with levofloxacin treatment was also effective in high *H*.* pylori *density. In sequential protocols, amoxicillin is administered before other antibiotics. Amoxicillin disrupts the bacterial cell wall of *H*.* pylori *and allows the antibiotics administered later to penetrate the bacteria. Due to the disrupted cell wall, *H*.* pylori* cannot form a reflux pump, and the effect of antibiotics can be preserved.^20^

According to a 2023 study in Türkiye, the overall resistance rates were as follows: clarithromycin 28.5%; metronidazole 44.8%; levofloxacin 23.1%.^21^ In the present study, the BQT regimen, which also included bismuth, did not show a favorable increase in eradication rates, but the eradication rate of BSLT was highly acceptable. Although the BQT results are in parallel with these studies, it was thought that metronidazole resistance might be responsible for the lower eradication rates in the BQT regimen.

Bismuth exerts a direct bactericidal effect on *H*.*pylori*.^21^ ^-22^ It is a well-known cytoprotective agent and, by increasing the Prostaglandin E2 (PGE2) level, it effectively protects gastric mucosa against pepsin. It also exerts a synergistic effect with antibiotics and may reduce the development of antibiotic resistance.^22^ Bismuth was also used in all 3 regimens used in the country due to treatment resistance.

Some studies have reported that increased *H*.* pylori* colonization density is associated with peptic ulcer formation and its complications.^23^ Moshkowitz et al^24^reported that favorable eradication rates were only achieved in patients with low pretreatment urease activity as assessed using urea breath tests (UBTs). In other studies, Lai et al^25^ and Shah et al^26^ investigated the relationship between histopathologic pretreatment *H*.* pylori* density and bacterial eradication and ulcer healing rates. They found a negative correlation between density and eradication rates, consistent with the results. All these results may suggest that more effective treatment options should be selected in patients with severe *H*.* pylori* colonization density. In the past study, it was also found that high bacterial loads in UBTs were negatively associated with the achievement of eradication with triple treatment. However, differences between groups were not significant in patients who received a quadruple eradication regimen in 2016.^27^ In the present study, it was observed that histopathologically, increased *H*.* pylori* density had a negative effect on treatment success in all 3 groups.

Studies on the CYP2C19 polymorphism in Türkiye have shown that the percentage of “poor” metabolizers is 1%-5%, and the percentage of “homozygous extensive” metabolizers is 75%-84%.^28^ In addition, due to the high rate of extensive metabolizers, it is suggested that esomeprazole and rabeprazole should be the preferred PPIs in Europe and North America.^29^ Rabeprazole was preferred in all groups of the study. In addition, vonoprazan is a new proton pump inhibitor that provides a sustained acid inhibitory effect and is approved for the treatment of gastroesophageal reflux disease, suggesting that increasing the pH levels in the stomach using vonoprazan may achieve an optimum eradication rate.^30^

Bismuth-containing sequential therapy with levofloxacin had a success rate of 81.3% in women and 91.5% in men, significantly higher for men than for women (*P* = .015). In other treatments, the results did not change according to gender. In this retrospective study, no demographic data that could explain this situation could be found. No publications on this topic in the literature were found. In prospective studies, treatment success could be re-evaluated based on gender.

This study had some strengths and weaknesses. Its strengths are that it had a significant number of patients from a single center and that *H*.* pylori* detection and post-treatment follow-up were investigated histopathologically. The main limitation of this study was its retrospective nature. Moreover, no antibiotic resistance was investigated in this study.

A bismuth-containing ST with levofloxacin regimen may be preferred for the first-line treatment of *H*.* pylori* infection. This regimen is particularly effective in cases of moderate and severe *H*.* pylori* colonization. Effective *H*.* pylori* eradication could prevent severe complications associated with this infection and potentially reduce the risk of gastric cancer. Future studies should focus on the long-term effects of these treatments and the potential development of antibiotic resistance.

## Figures and Tables

**Table 1.** Treatment Groups

Group 1 (BQT)	Bismuth quadruple therapy: 14 days Tetracycline 500 mg qid Metronidazole 500 mg qid Bismuth subsalicylate 265 mg qid Rabeprazole 20 mg bid
Group 2 (BSTC)	Sequential therapy with clarithromycin and bismuth: First 7 days Amoxicillin 1000 mg bid Rabeprazole 20 mg bid Bismuth subsalicylate 265 mg qid Second 7 days Clarithromycin 500 mg bid Metronidazole 500 mg bid Rabeprazole 20 mg bid Bismuth subsalicylate 265 mg qid
Group 3 (BSTL)	Sequential therapy with levofloxacin and bismuth: First 7 days Amoxicillin 1000 mg bid Rabeprazole 20 mg bid Bismuth subsalicylate 265 mg qid Second 7day Levofloxacin 500 mg bid Rabeprazole 20 mg bid Bismuth subsalicylate 265 mg qid

BQT, bismuth-containing quadruple therapy; BSTC, bismuth-containing sequential therapy with clarithromycin; BSTL, bismuth-containing sequential therapy with levofloxacin.

**Table 2.** Eradication Rates of Treatment Regimens

Treatment Regimen	Successful Eradication (n)	Total (n)	Eradication Rate (%)
BSTC	157	210	74.80
BQT	163	218	74.80
BSTL	256	300	85.30

BQT, bismuth-containing quadruple therapy; BSTC, bismuth-containing sequential therapy with clarithromycin; BSTL, bismuth-containing sequential therapy with levofloxacin.

**Table 3.** Eradication Rates of Regimens According to *Helicobacter* *pylori* Density

*H*. *pylori* Intensity	Treatment Regimen	Successful Eradication	Total (n)	Eradication Rate (%)
Low	BSTL	43	56	76.8
	BQT	39	46	84.3
	BSTC	25	32	78.1
Intermediate	BSTL	73	86	84.9
	BQT	64	88	72.7
	BSTC	47	71	66.2
High	BSTL	140	158	88.6
	BQT	60	84	71.4
	BSTC	157	210	74.8

BQT, bismuth-containing quadruple therapy; BSTC, bismuth-containing sequential therapy with clarithromycin; BSTL, bismuth-containing sequential therapy with levofloxacin.

## Data Availability

The data that support the findings of this study are available on request from the corresponding author.
